# The pathogenesis-related protein PR-4b from *Theobroma cacao* presents RNase activity, Ca^2+^ and Mg^2+^ dependent-DNase activity and antifungal action on *Moniliophthora perniciosa*

**DOI:** 10.1186/1471-2229-14-161

**Published:** 2014-06-11

**Authors:** Sara Pereira Menezes, Edson Mario de Andrade Silva, Eline Matos Lima, Aurizângela Oliveira de Sousa, Bruno Silva Andrade, Livia Santos Lima Lemos, Karina Peres Gramacho, Abelmon da Silva Gesteira, Carlos Priminho Pirovani, Fabienne Micheli

**Affiliations:** 1 Departamento de Ciências Biológicas (DCB), Centro de Biotecnologia e Genética (CBG), Universidade Estadual de Santa Cruz (UESC), Rodovia Ilhéus-Itabuna, km 16, 45662-900 Ilhéus, BA, Brazil; 2 Universidade Estadual do Sudoeste da Bahia (UESB), Av. José Moreira Sobrinho, Jequié, Bahia 45206-190, Brazil; 3 Cocoa Research Center, CEPLAC/CEPEC, 45600-970 Itabuna, BA, Brazil; 4 Departamento de Biologia Molecular, Embrapa Mandioca e Fruticultura, Rua Embrapa, s/n°, CEP44380-000 Cruz das Almas, Bahia, Brazil; 5 CIRAD, UMR AGAP, F-34398 Montpellier, France

**Keywords:** Nucleases, Gene expression, ROS production, Molecular modeling

## Abstract

**Background:**

The production and accumulation of pathogenesis-related proteins (PR proteins) in plants in response to biotic or abiotic stresses is well known and is considered as a crucial mechanism for plant defense. A pathogenesis-related protein 4 cDNA was identified from a cacao-*Moniliophthora perniciosa* interaction cDNA library and named *TcPR-4b*.

**Results:**

TcPR-4b presents a Barwin domain with six conserved cysteine residues, but lacks the chitin-binding site. Molecular modeling of TcPR-4b confirmed the importance of the cysteine residues to maintain the protein structure, and of several conserved amino acids for the catalytic activity. In the cacao genome, TcPR-4b belonged to a small multigene family organized mainly on chromosome 5. *TcPR-4b* RT-qPCR analysis in resistant and susceptible cacao plants infected by *M. perniciosa* showed an increase of expression at 48 hours after infection (hai) in both cacao genotypes. After the initial stage (24-72 hai), the *TcPR-4b* expression was observed at all times in the resistant genotypes, while in the susceptible one the expression was concentrated at the final stages of infection (45-90 days after infection). The recombinant TcPR-4b protein showed RNase, and bivalent ions dependent-DNase activity, but no chitinase activity. Moreover, TcPR-4b presented antifungal action against *M. perniciosa*, and the reduction of *M. perniciosa* survival was related to ROS production in fungal hyphae.

**Conclusion:**

To our knowledge, this is the first report of a PR-4 showing simultaneously RNase, DNase and antifungal properties, but no chitinase activity. Moreover, we showed that the antifungal activity of TcPR-4b is directly related to RNase function. In cacao, TcPR-4b nuclease activities may be related to the establishment and maintenance of resistance, and to the PCD mechanism, in resistant and susceptible cacao genotypes, respectively.

## Background

The production and accumulation of pathogenesis-related proteins (PR proteins) in plants in response to biotic or abiotic stresses is well known and is considered as a crucial mechanism for plant defense [1-4]. The PR proteins are defined as plant proteins induced in pathological situations not necessary implied with a direct interaction with the pathogen [4,5]. However, several characterized PR proteins showed enzymatic activities related to antimicrobial properties. Among the 17 PR protein families already described, at least 9 present enzymatic activity such glucanases (PR-2; [6]), osmotins and thaumatins (PR-5; [7-10]), protease inhibitors (PR-6; [11]), lysozymes (PR-8; [12]), peroxidase (PR-9; [13,14]), ribonucleases (PR10; [15-17]) and chitinases (PR-3, PR-4, PR-8, PR-11; [18-20]). Focusing on PR-4 proteins, several studies showed the involvement of this family in plant defense responses regulated by signal molecules, such as salicylic acid (SA), abscissic acid (ABA), jasmonate (JA) and ethylene (ET). In rice, maize and wheat, the *PR-4* gene expression was induced in the first hours (until 96 h) after the contact with the pathogenic fungus and/or the fungal elicitors (e.g. moniliformin) [1,2,21]. Moreover, it has been shown that the activation of wheat *PR-4* genes depends on both SA and JA dependent metabolic pathways [22]. In dicotyledones, the induction of the *PR-4* gene expression was also observed in the initial stages of disease in the case of red pepper infected by the *Pepper mild mottle virus*[23] or tobacco infected by the *Tabacco mosaic virus*[24], as well as in response to ethephon – an ET-releasing compound in Chinese cabbage [25]. In apple, the *MdPR-4* gene expression was associated to the plant defense response against *Botryosphaeria dothidea*, through signalization pathways dependent of SA and JA, as well as to several physiological functions such as flower formation [26].

The presence of a C-terminal conserved Barwin domain – first identified in proteins obtained from barley seeds, and containing 6 conserved cystein residues forming disulfide bridges [27] – corresponds to the characteristic structure of the PR-4 proteins. Moreover, most of the PR-4 proteins have a signal peptide and some of them show transmembrane structure in the N-terminal region [28-30]. Some PR-4 proteins also present a C-terminal extension domain involved in protein targeting to the vacuole [31-33]. It has been shown that the barley Barwin protein is able to slightly interact with the oligosaccharide β-(1,4) tetramer of N-acetylglucosamine, an analog of chitin [27,34]. The PR-4 protein classification is based on the presence (class I) or absence (class II) of the chitin ligation domain [35] also known as hevein-like domain – from the name of hevein, a small antifungal molecule present in *Hevea brasilienses* latex [36]. The PR-4 proteins could show chitinase, RNase and/or DNase activities as well as antifungal properties; these functions could be related (or not) one to another and have been detected in both class I and II proteins. The class I tobacco PR-4 protein NtCBP20 showed antifungal activity against *Trichoderma viride* and *Fusarium solani* by causing germinative tube lyses and fungal growth inhibition [37]. In fig, the class I FaPR-4 protein as well as its truncated form FaPR-4c – lacking the N-terminal region to mimic a class II PR-4 – showed RNase, chitinase and antifungal activity [33]. However, some PR-4 only present RNase and antifungal activities [26,32,38] or RNase and DNase activity but no chitinase property [35]. These characteristics may also be observed in multigene family members; in wheat, four class II PR-4 (named wheatwin 1 to 4) were isolated, and all of them showed antifungal activity [39]. Moreover the recombinant protein wheatwin1 showed RNase activity, and a site-directed mutagenesis experiment revealed that the reduction of RNase activity was correlated to the loss of wheatwin1 antifungal properties – analyzed on mycelial growth [18].

Despite the importance of the PR proteins in plant defense, only few members of this family, TcPR-1, TcPR5 and TcPR-10, have been previously fully characterized in *Theobroma cacao*[40-43]. Two TcPR-1, named TcPR-1f and TcPR-1g, were identified and analyzed at phylogenetic and expression levels. The *TcPR-1g* gene was up-regulated during the biotrophic stage of the infection of susceptible cacao genotype by the basidiomycete *Moniliophthora perniciosa*[42] – causal agent of the witches’ broom disease, one the most devastating diseases of cacao trees [44]. The *TcPR5* gene encodes an osmotin-like protein identified in leaves and roots from cacao ‘Comun’ (Lower Amazon Amelonado type) sumitted to drought, indicating its involvement in tolerance to osmotic stress [43]. In the case of TcPR-10, this gene was related to defense responses in the final stages of the witches’ broom disease. The corresponding TcPR-10 protein also presents RNase enzymatic activity and antifungal action against *M. perniciosa* and *Saccharomyces cerevisiae*[40,45]. Here, we described another PR protein, the TcPR-4b, which was identified from a cacao pod-*M. perniciosa* interaction cDNA library (Ceplac project/unpublished data) and from a cacao meristem-*M. perniciosa* interaction library [46]. First *in silico* and semi-quantitative RT-PCR analysis showed that PR4-b genes from cacao were differentially expressed between resistant and susceptible cacao varieties submitted to *M. perniciosa* infection [46]. The characterization of the TcPR-4b and the cacao PR-4 family has been facilitated by the recent publication of the *T. cacao* genome [47]. Using bioinformatics, molecular biology and biochemistry, we showed that *TcPR-4b* belonged to a small multigene family and was differentially expressed in resistant and susceptible cacao genotypes infected *vs* non infected by *M. perniciosa*. Moreover, to our knowledge, this is the first report of a plant PR-4 that presents both RNase, DNase and antifungal properties but no chitinase activity. This work also describes for the first time a complete PR-4 gene family in cacao, an important crop that may be used as plant model of fruit tree according to recent studies [47]. Finally, our paper shows that TcPR-4b may be a good candidate for biotechnological or molecular genetics control strategies of the witches’ broom disease in cacao.

## Results

### Sequence analysis of TcPR-4b

**Figure 1:**
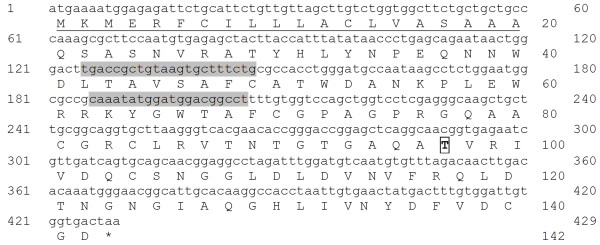
**Nucleotide and amino acid sequences of TcPR-4b.** The signal peptide is underlined; the remaining sequence corresponds to the Barwin domain. The asterisk represents the ORF termination codon. The putative phosphorylation site is squared on the amino acid sequence. RT-qPCR primer position is indicated in gray.

**Figure 2:**
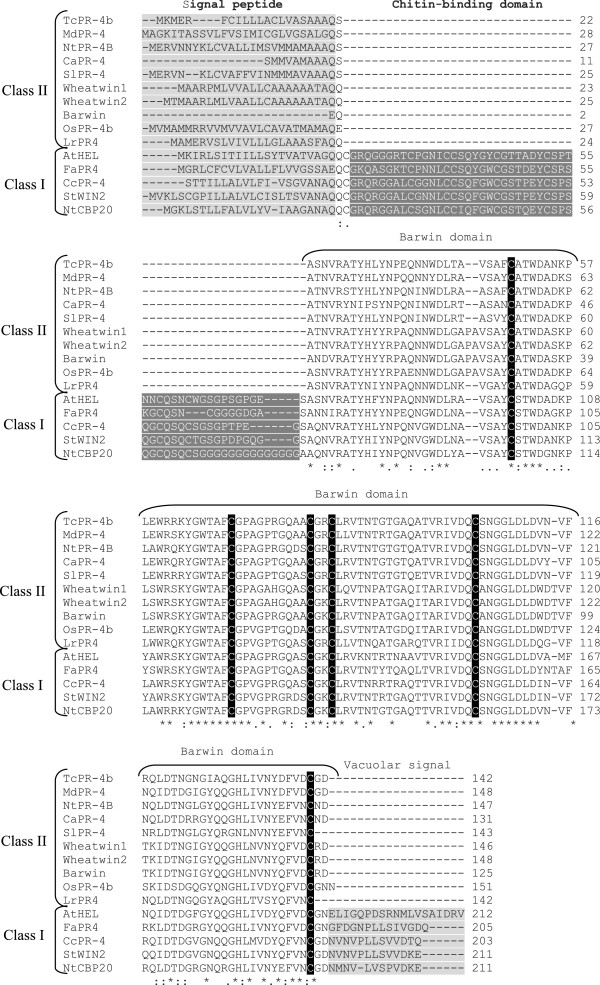
**Amino acid sequence alignment of TcPR-4b with other PR4 proteins identified from NCBI database.** The class I PR4s used for alignment are: AtHEL (*Arabdopsis thaliana,* NP_187123.1), FaPR4 (*Ficus pumila* var. awkeotsang, ADO24163.1), CcPR-4 (*Capsicum chinense,* BAD11073), StWIN2 (*Solanum tuberosum*, P09762), NtCBP20 (*Nicotiana tabacum*, AAB29959.2). The class II PR-4 s used for the alignment were: MdPR-4 (*Malus domestica*, AFH74426.1), NtPR-4B (*Nicotiana tabacum*, P29063.1), CaPR-4 (*Capsicum annuum*, AAF63520.1), SlPR-4 (*Solanum lycopersicum*, NP_001234083.1), Wheatwin1 (*Triticum aestivum,* O64392.1), Wheatwin2 (*Triticum aestivum,* O64393.1), Barwin (*Hordeum vulgare,* 1BW3_A), OsPR-4b (*Oryza sativa*, AY435041), LrPR4 (*Lycoris radiate,* ACI31201.1). Gaps introduced to get the best alignment are indicated by (-), (*) represents identical amino acids between all sequences, (.) and (:) represent conserved substitutions and semi-conserved substitutions, respectively. Signal peptide, chitin-binding domain and vacuolar signal are indicated in gray scale. The Barwin domain is also indicated; the six conserved cysteines in the Barwin domain are highlighted in black.

**Figure 3:**
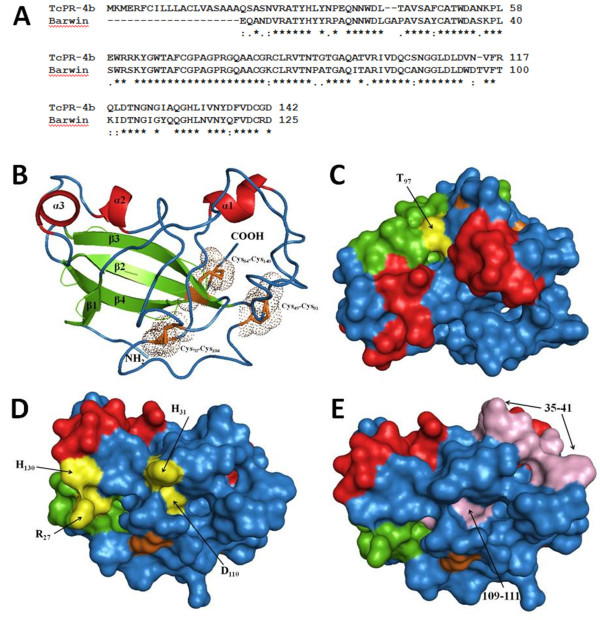
**Tridimensional structure of TcPR-4b obtained by homology modeling with the barley Barwin protein (PDB code 1BW3_A.pdb) as template using SWISS-MODEL. A**. Alignment of TcPR-4b with the barley Barwin protein. **B**. Ribbon representation of the TcPR-4b structure. The secondary structures are indicated by different colors: α-helices in red, β-strands in green and P-loops in blue. Dots indicate conserved cysteine residues forming disulfide linkages C_49_-C_81_, C_70_-C_104_ and C_84_-C_143_. **C**. Molecular surface of TcPR-4b with the T_97_ putative phosphorylation site indicated in yellow. **D**. Molecular surface position of conserved amino acids H_31_, H_130_, D_110_ e R_27_ which are highlighted in yellow. **E**. Molecular surface position of the regions 35-41 and 109-111 are indicated in pink.

**Figure 4:**
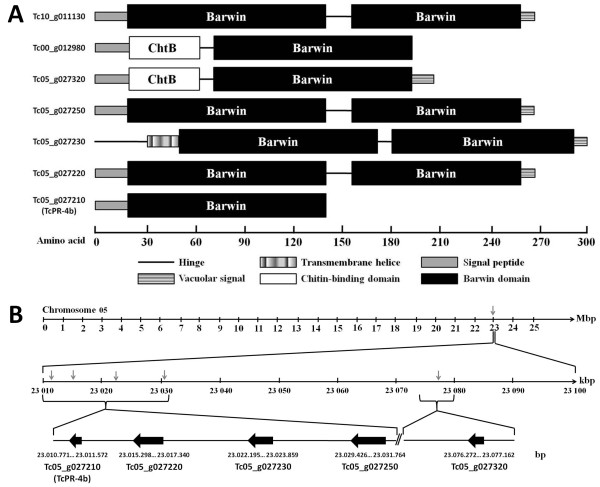
**Scheme of the PR-4 family in the *Theobroma cacao *genome database (CocoaGenDB). A**. Struture of the PR-4 proteins from *Theobroma cacao*. ChtB: chitin-binding domain. **B**. Localization and organization of five of the PR-4 genes on *Theobroma cacao* chromosome 5. Grey arrows indicate the PR-4 gene position.

The *TcPR-4b* gene identified from *T. cacao-M. perniciosa* interaction library presents an ORF of 429 nucleotides encoding a protein of 142 amino acid residues (Figure 1). A hydrophobic region was observed at the N-terminal of the protein which may correspond to a transmembrane N-terminal helix or a signal peptide [48]. A detailed analysis using the TMHMMA server 2.0 did not reveal any transmembrane helix and the SignalP 4.0 Server predicted a signal peptide with a putative cleavage site between the A_20_ and Q_21_ (Figure 1). The TcPR-4b protein with signal peptide has a putative molecular mass of 15.43 kDa and a putative pI of 6.69, while the protein without signal peptide has a molecular mass of 13.29 kDa and a putative pI of 6.06. Only one phosphorylation site (T_97_) was observed (Figure 1). No glycosylation and acetylation site was found in the TcPR-4b sequence (data not shown). The TcPR-4b contains the functional Barwin domain (PF00967, E-value 1.8.10^-65^) (Figure 2). The TcPR-4b amino acid sequence presents six conserved cystein residues (C_49_, C_70_, C_81_, C_84_, C_104_, C_140_) characteristic of the Barwin domain and responsible for disulfide bond formation (Figures 2 and 3B). TcPR-4b does neither present chitin-binding domain nor C-terminal conserved domain – which corresponds to a signal for protein addressing to the vacuole [31,35] (Figure 2). The analysis of the TcPR-4b domains by comparison with other class I and PR-4s from various mono- and dicotyledonous species, showed that TcPR4-b presents a high identity with class II PR-4s from other plant species (Figure 2; Table 1) such as *Malus domestica* (86%; MdPR-4 accession number AFH74426.1), *Nicotiana tabacum* (84%; NtPR-4B accession number P29063.1), *Solanum lycopersicum* (78%; SlPR-4 accession number NP_001234083.1), *Capsicum annuum* (77%; CaPR-4 accession number AAF63520.1), *Capsicum chinense* (78%; CcPR-4 accession number BAD11073), *Triticum aestivum* (75%; Wheatwin2 accession number O64393.1) and *Lycoris radiate* (75%; LrPR4 accession number ACI31201.1). The blastn analysis of TcPR-4b against the CocoaGenBD from *T. cacao*, detected a single gene located on the chromosome 5 and showing 100% of identity with TcPR-4b. The complete *TcPR-4b* gene sequence (including UTRs and ORF) was 802 bp in length and contained 2 exons of 171 and 258 bp, and 1 intron of 82 bp (Additional file 1). tblastn search in the CocoaGenBD also revealed the presence of 6 more PR-4 present in the *T. cacao* genome (Figure 4 and Additional file 2). Four of them (Tg10_g011130, Tg05_g027250, Tg05_g027230 and Tg05_g027220) showed 2 Barwin domains, two of them (Tg05_g012980 and Tg05_g027320) contained one Barwin and one chitin-binding domains, and one protein sequence (TcPR-4b) showed a unique Barwin domain (Figure 4A). Only one PR-4 protein from cacao (Tg05_g027230) presented a transmembrane helix at the N-terminal end of the protein while the others sequences, including TcPR-4b, presented a signal peptide (Figure 4A). Except for Tg05_g012980 and TcPR-4b, the PR-4 proteins from cacao contained a vacuolar signal (Figure 4A). The PR-4 proteins from *T. cacao* presented 55.4 to 78.69% of identity with TcPR-4b (Additional file 2). Five of the 7 cacao *PR-4* genes (TcPR-4b, Tc05_g027320, Tc05_027220, Tc05_g027250 and Tc05_g027230) were located on the chromosome 5, one on the chromosome 10 (Tc10_g011130) and the last one (Tc00_t012980) did not present a defined localization (chromosome 0; Additional file 2). The analysis of the chromosome 5 structure showed that the 5 PR-4 genes were organized in tandem in the (-) strand of the chromosome in a region corresponding approximately to 0.3% (from 23010 to 23100 Kbp) of the total chromosome length (Figure 4B).

**Table 1:** Characteristics of PR-4 from different plant species in comparison with TcPR-4b

**Name**	**Specie**	**Class**	**Identity with TcPR-4b (%)**	**E-value**	**Structure**	**Activity**				**Acession number**	**Reference**
						**Chitinase**	**RNase**	**DNase**	**Antifungal**		
TcPR-4b	*Theobroma cacao*	II	-	-	SP-Barwin Domain	No	Yes	Yes	Yes	FC072496.1; ES440503.1	This study
CcPR-4	*Capsicum chinense*	II	72	-	SP-Barwin Domain	No	Yes	Yes	nd	nd	[35]
CcPR-4	*Capsicum chinense*	I	78	5.10^-62^	SP-ChtBD-Barwin Domain-VS	nd	nd	nd	nd	BAD11073	[23]
LrPR4	*Lycoris radiate*	II	75	1.10^-58^	SP-Barwin Domain	nd	Yes	No	Yes	ACI31201.1	[38]
OsPR-4b	*Oryza sativa*	II	69	-	SP- Barwin Domain	nd	nd	nd	Yes	AY435041	[29]
MdPR-4	*Malus domestica*	II	86	2.10^-69^	SP-Barwin Domain	nd	Yes	nd	Yes	AFH74426.1	[26]
Wheatwin1	*Triticum aestivum*	II	69	-	SP-Barwin Domain	nd	Yes – inhibition by 5′ADP	nd	Yes	O64392.1	[39]
Wheatwin2	*Triticum aestivum*	II	75	2.10^-56^	SP-Barwin Domain	nd	nd	nd	Yes	O64393.1	[39,49]
FaPR4	*Ficus awkeotsang*	I	78	6.10^-61^	SP-ChtBD-Barwin Domain-VS	Yes	Yes – type A inhibited with 5′ADP	No	Yes	ADO24163.1	[33]
FaPR4 modified^a^	*Ficus awkeotsang*	II	65	6.10^-61^	SP -Barwin Domain-VS	Yes	Yes – type A inhibited with 5′ADP	No	Yes	ADO24163.1	[33]
NtCBP20	*Nicotiana tabacum*	I	81	8.10^-65^	SP-ChtBD-Barwin Domain-VS	Yes – when together with β-1,3 glucanase	nd	nd	Yes	AAB29959.2	[37]
AtHEL modified^a^	*Arabdopsis thaliana*	I	27	3.10^-61^	ChtBD	No	No	nd	Yes	NP_187123.1	[32]
AtHEL modified^a^	*Arabdopsis thaliana*	II	77	3.10^-61^	Barwin Domain	No	Yes	No	Yes	NP_187123.1	[32]
NtPR-4B	*Nicotiana tabacum*	II	84	2.10^-67^	SP-Barwin Domain	nd	nd	nd	nd	P29063.1	[30]
CaPR-4	*Capsicum annuum*	II	77	3.10^-59^	SP-Barwin Domain	nd	nd	nd	nd	AAF63520.1	[50]
SlPR-4	*Solanum lycopersicum*	II	78	1.10^-61^	SP-Barwin Domain	nd	nd	nd	nd	NP_001234083.1	[24]
StWIN2	*Solanum tuberosum*	I	77	1.10^-61^	SP-ChtBD-Barwin Domain-VS	nd	nd	nd	nd	P09762	[28]
Barwin	*Hordeum vulgare*	II	74	7.10^-58^	Barwin Domain	nd	nd	nd	nd	1BW3_A	[27]

nd: non determined; SP: signal peptide; ChtBD: chitin-binding domain; VS: vacuolar signal.

^a^Protein modified by genetic engineering.

### Phylogeny of T. cacao PR-4

**Figure 5:**
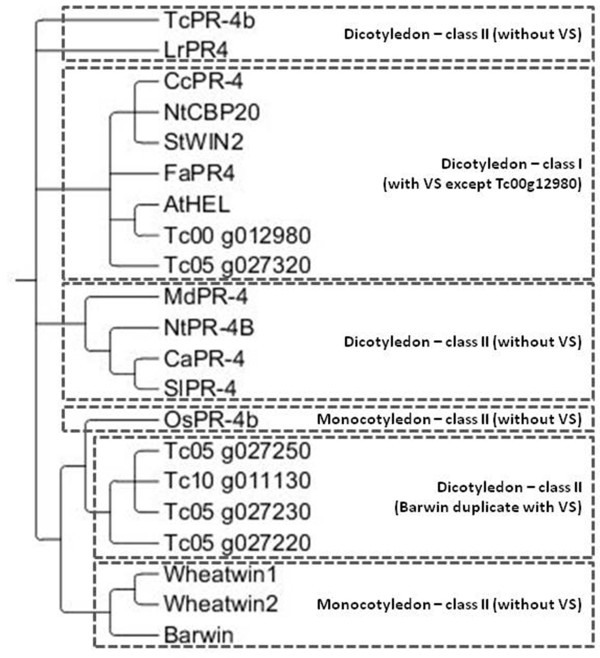
**Bayesian phylogenetic analysis, using amino acid data.** Bayesian consensus phylogram of PR-4 from *T. cacao* and other plant species. VS: vacuolar signal.

The class I PR-4 proteins were grouped in a unique clade, and except for Tc00g_12980, all the proteins had a vacuolar signal (Figure 5). The class II proteins from dicotyledons as well as monocotyledons had no vacuolar signal, except for the cacao proteins that contain 2 Barwin domains; these last ones (Tg10_g011130, Tg05_g027250, Tg05_g027230 and Tg05_g027220) formed a separated clade. The TcPR-4b protein had a higher identity with the class I sequences Tg05_g012980 (78%) and Tg05_g027320 (68%) (Additional file 2). *TcPR-4b* is also closer to Tg05_g012980 in relation to the genic organization; one and no introns were identified for TcPR-4b and Tg05_g012980, respectively (Additional file 2). In the phylogenetic analysis, the protein TcPR-4b is closer to class I than from class II proteins with two Barwin domains (Figure 5).

### Molecular modeling of TcPR-4b

The alignment of the amino acid sequence of TcPR-4b with the barley Barwin protein (1BW3_A.pdb) presented 74.4% of identity and 89% of similarity (E-value 1.43.10^-45^; Figure 3A), and a RMSD of 0.092 Å. Identity above 50% and E-value below 4.10^-43^ indicate that the Barwin protein is a good model to be used as a template. The molecular modeling of TcPR-4b (without the signal peptide) showed i) 3 α-helices, α1 (37-42), α2 (95-98) and α3 (103-107); ii) 4 β-sheets, β1 (6-8), β2 (62-68), β3 (72-79) and β4 (110-117); and iii) 8 loops L1 (1-5), L2 (9-36), L3 (43-61), L4 (69-71), L5 (80-94), L6 (99-102), L7 (108-109) and L8 (118-121) (Figure 3B). The validation analysis (Ramachandran plot) of the TcPR-4b model presented 73.8% of residues in most favored regions, 25.2% in additional allowed regions and only 1% in disallowed regions indicating that 99% of amino acid residues are located in favored regions (Additional file 3). In addition, PSIPRED validation revealed reasonable secondary structure, and ANOLEA showed good energy values. The conservation of the cysteins along the PR-4 polypeptides (Figure 2) highlights the importance of these residues to maintain the tridimensional structure of the protein [51]. In the TcPR-4b, the cystein residues formed three disulfide bonds C_49_-C_81_, C_70_-C_104_ and C_84_-C_140_ (Figure 3B). The phosphorylation site T_97_ is located in a β-sheet, on the molecule superficies, i.e. in a region accessible to addition of a phosphate group (Figure 3C). In PR-4s, the conserved H_11_, H_113_, D_92_ and R_7_ are considered to be critical for antifungal and ribonuclease activity [18,39]. In TcPR-4b the conserved residues H_31_, H_130_, D_110_ and R_27_ were observed; H_31_ and D_110_ are located in loops – D_110_ being more internal than H_31_ located at the surface of the protein (Figure 3D). The amino acids H_130_ and R_27_ formed a β-sheet at the molecular surface, i.e. in an accessible region with moderate flexibility (Figure 3D). The molecular modeling of TcPR-4b also revealed the presence of a conserved region 35-41/PEQNNWD located in a loop exposed on the molecule surface, i.e. in accessible region with high flexibility (Figure 3E). On the contrary, the region 109-111/LDL, which also formed a loop, was located in the internal part of the protein (Figure 3E).

### Expression of TcPR-4b in resistant and susceptible Theobroma cacao genotypes

**Figure 6:**
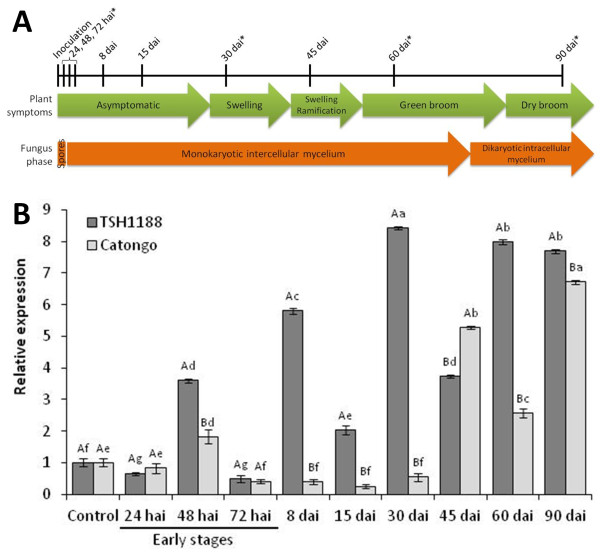
**Relative expression of *TcPR-4b *in TSH1188 and Catongo meristems inoculated or not (control) with *M. perniciosa*. A**. Representation of the plant symptoms and fungus phase during the infection time course in Catongo genotype. The harvesting times of inoculated plants are indicated on the top of the figure. (*) indicates the times that were harvested also in the non-inoculated (control) plants. **B**. RT-qPCR of *TcPR4b*. The control, used as calibrator (for this reason is always 1), corresponds to the average of the expression values of *TcPR-4b* in 5 non-inoculated samples in each genotype (see also Methods section). The results are the arithmetical mean of the repetitions ± standard error. Different letters indicate significant statistical difference between samples by the Scott-Knott test (P ≤ 0.01): lower case letters correspond to statistics between harvesting times for each genotype while upper case letters correspond to statistics between genotypes for each harvesting time. dai: days after inoculation; hai: hours after inoculation.

The expression of the *TcPR-4b* gene was analyzed in two cacao genotypes, TSH1188 (resistant to witches’ broom disease) and Catongo (susceptible) infected or not (control) with *M. perniciosa* (Figure 6A and B). For both genotypes and for all the harvesting point, the PCR amplification occurred at the same and unique melting temperature showing that only the TcPR-4b gene was amplified (Additional file 4). In both genotypes, the *TcPR-4b* expression was low at 24 hai, increased at 48 hai (3.6 and 1.86 times more than the control for TSH1188 and Catongo, respectively) and then decreased at 72 hai (Figure 6B). At 48 hai, the *TcPR-4b* expression was about 2 times higher in TSH118 than in Catongo. From 8 to 30 dai, *TcPR-4b* was repressed in Catongo and overexpressed in TSH1188 in comparison to the control; the expression in TSH1188 was about 12, 6 and 17 times higher than the one observed in Catongo at 8, 15 and 30 dai, respectively (Figure 6B). From 45 to 90 dai, TcPR-4b was superexpressed in both genotypes in comparison to the control (about 8 times more at 60 and 90 dai). Except at 45 dai, the expression was higher in TSH1188 than in Catongo (3.2 and 1.2 times at 60 and 90 dai, respectively). At 45 dai, the *TcPR-4b* expression was about 1.34 higher in Catongo than in TSH1188.

### Production of the recombinant TcPR-4b protein

**Figure 7:**
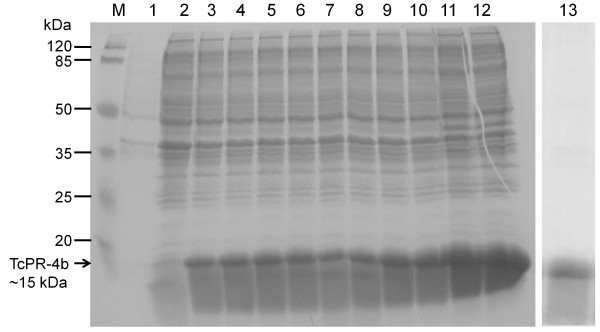
**SDS-PAGE analysis of the recombinant TcPR-4b protein.** M. Molecular weight marker (Thermo Scientific). Lane 1: pET28a without insert and without induction. Lane 2: pET28a without insert and 4 h after induction with 1 M of IPTG at 37°C. Lane 3: pET28a-*TcPR-4b* 1 h after induction with 1 M of IPTG at 37°C. Lane 4: pET28a-*TcPR-4b* 2 h after induction with 1 M of IPTG at 37°C. Lane 5: pET28a-*TcPR-4b* 3 h after induction with 1 M of IPTG at 37°C. Lane 6: pET28a-*TcPR-4b* 4 h after induction with 1 M of IPTG at 37°C. Lane 7: pET28a-*TcPR-4b* 1 h after induction with 0.4 M of IPTG at 37°C. Lane 8: pET28a-*TcPR-4b* 2 h after induction with 0.4 M of IPTG at 37°C. Lane 9: pET28a-*TcPR-4b* 3 h after induction with 0.4 M of IPTG at 37°C. Lane 10: pET28a-*TcPR-4b* 4 h after induction with 0.4 M of IPTG at 37°C. Lane 11: pET28a-*TcPR-4b* after induction overnight with 1 M of IPTG at 18°C. Lane 12: pET28a-*TcPR-4b* after induction overnight with 0.4 M of IPTG at 18°C. Lane 13: purified insoluble fraction of the recombinant TcPR-4b (15.43 kDa). The arrow indicates the recombinant TcPR-4b protein.

The *TcPR-4b* gene was cloned in pET28a plasmid and was successfully expressed in *Escherichia coli* Rosetta (Figure 7, lanes 3 to 12) while no visible band was observed in the controls (pET28a without insert in presence or not of IPTG) (Figure 7, lanes 1 and 2). The induction of the protein expression was higher at 18°C than at 37°C (regardless of the IPTG concentration and the incubation period; Figure 7, lanes 11 and 12). For this reason, the conditions of 0.4 M of IPTG and 18°C (overnight) were established as the best conditions of TcPR-4b induction. The recombinant protein had a molecular mass of 15 kDa, close to the one predicted (15.43 kDa with His-Tag). The TcPR-4b was successfully purified with a Talon resin metal-affinity column (Figure 7, lane 13).

### Ribonuclease and Ca^+2^ and Mg^+2^ dependent deoxyribonuclease activities

**Figure 8:**
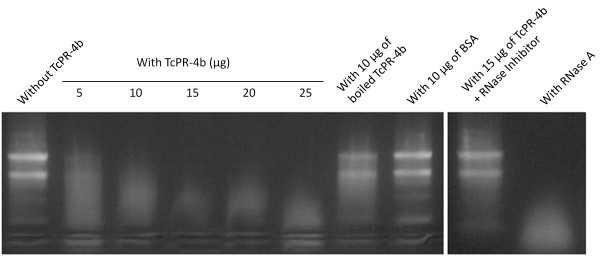
**Ribonuclease activity of the recombinant TcPR-4b on tomato (*Solanum lycopersicum *var. Micro-Tom) total RNA (5 μg).** The incubation with TcPR-4b was carried out for 30 min at 25°C. The boiling conditions were 10 min at 95°C. The RNase inhibitor was the RiboLock (40 U; Thermo Scientific). The incubation conditions of the RNase A (Thermo Scientific) were 10 min at 25°C.

**Figure 9:**
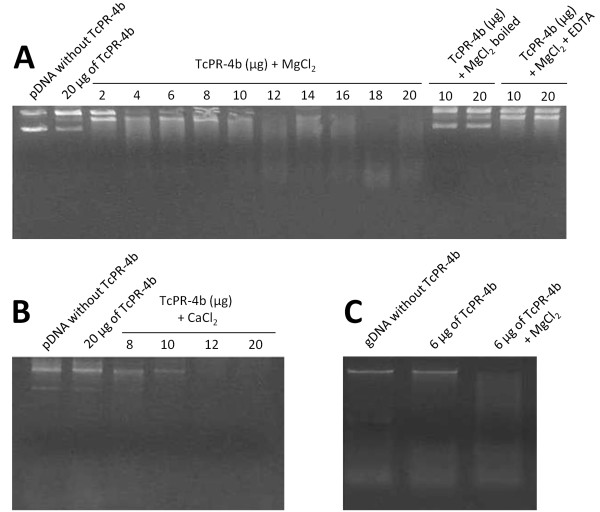
**Deoxyribonuclease activity of recombinant TcPR-4b. A** and **B**. DNase activity of the TcPR-4b tested against 1 μg of plasmidial DNA (pGEM-T® Easy Vector; Promega). **C**. DNase activity of the TcPR-4b tested against 1 μg of genomic DNA from *Nicotiana tabacum*. The incubation with TcPR-4b was made overnigth at 25°C. The boiling conditions were 10 min at 95°C. The following concentrations were used: 10 mM of MgCl_2_, 10 mM of EDTA; 1 mM CaCl_2_. gDNA: genomic DNA; pDNA: plasmidial DNA.

The nuclease function of the purified recombinant TcPR-4b was analyzed by incubation of the protein with RNA or DNA and visualization of the nucleic acid degradation patterns on agarose gels (Figures 8 and 9). Tomato RNA degradation (smears of RNA) was observed after 30 min of incubation with TcPR-4b; the degradation was more important when the amount of the purified recombinant protein increased (Figure 8; 5 to 25 μg of the protein). The RNA degradation pattern in the presence of 25 μg of TcPR-4b was similar to the positive control degradation obtained in samples incubated with commercial RNase A (Figure 8; 25 μg of the TcPR-4b protein and with RNase A). The negative controls showed no degradation (samples without TcPR-4b and with 10 μg of BSA instead of TcPR-4b) or very slight degradation smear (with 10 μg of boiled TcPR-4b; Figure 8). Moreover, no degradation was observed in presence of 15 μg of TcPR-4b plus RNase inhibitor (that inhibits RNase A, B and C), corroborating that the recombinant TcPR-4b had a ribonuclease activity. Plasmidial as well as genomic DNA degradation was observed after overnight incubation with TcPR-4b plus 10 mM MgCl_2_ (Figure 9A and C) or 1 mM of CaCl_2_ (Figure 9B). No degradation was observed in presence of TcPR-4b without MgCl_2_ or CaCl_2_ (Figure 9A, B and C). The degradation was more important when the amount of TcPR-4b increased (Figure 9A and B). The negative controls (sample without TcPR-4b and with TcPR-4b plus MgCl_2_ boiled sample) showed no degradation (Figure 9A, B and C). Samples of TcPR-4b plus MgCl_2_ incubated with 10 mM of EDTA (chelator of various metal ions), showed no DNase activity (Figure 9A). No RNA or DNA degradation was observed using the extract of bacteria containing the pET28 vector without insert (negative control), avoiding the possible action of some bacterial component on the obtained results (Additional file 5A and B).

### Chitinase activity

**Figure 10:**
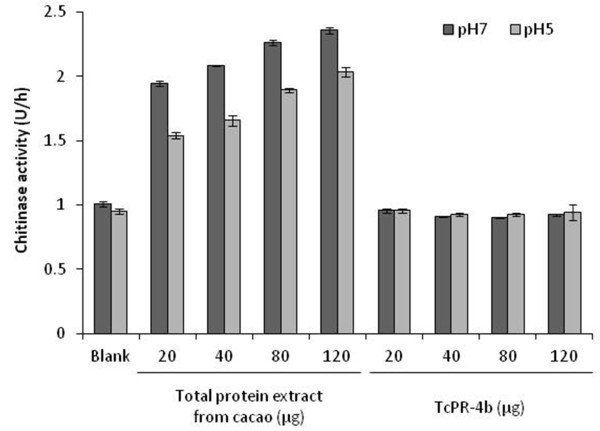
**Chitinase activity of TcPR-4b.** Blank: buffer A or B (see Methods section).

The chitinase activity of TcPR-4b was tested in two different conditions of pH using the bufferA/pH 5.0 and buffer B/pH 7.0 (see Methods section). Regardless of the buffer used, TcPR-4b did not show any activity in comparison to the blank (buffer A or B), while the protein extract from cacao meristems presented a chitinase activity between 1.5 to 2.5 U/h depending of the buffer and protein concentration used (Figure 10). Moreover, the TcPR-4b amount (from 20 μg to 120 μg) did not affect the results (Figure 10).

### Antifungal activity of TcPR-4b

**Figure 11:**
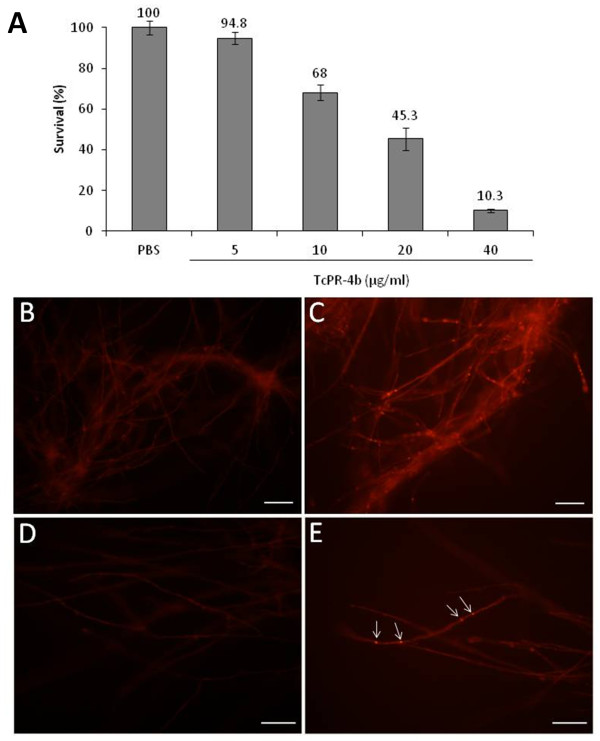
**Action of TcPR-4b on dikaryotic *M. perniciosa *survival. A**. Antifungal activity of recombinant TcPR-4b protein. PBS: phosphate buffered saline. **B** to **E**. ROS detection on dikaryotic *M. perniciosa* broken hyphae in absence (**B** and **C**; control -PBS) or in presence (**C** and **E**) of TcPR-4b (10 μg/ml). **B** and **C**, bars = 30 μm; **D** and **E**, bars = 10 μm. The arrows indicated the fluorescent foci.

**Figure 12:**
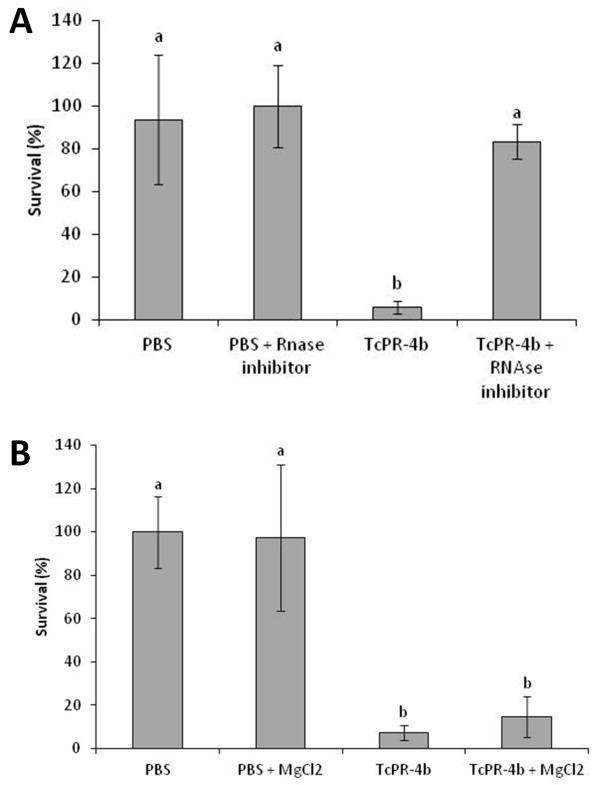
**Action of TcPR-4b on dikaryotic *M. perniciosa *survival in relation to RNase and DNase activity. A**. Action of TcPR-4b on dikaryotic *M. perniciosa* survival in presence of RNase inhibitor. The following concentrations were used: 40 μg/ml of TcPR-4b and 800 U of RNase inhibitor. **B**. Action of TcPR-4b on dikaryotic *M. perniciosa* survival in presence of MgCl_2_. The following concentrations were used: 40 μg/ml of TcPR-4b and 10 mM of MgCl_2_.

To test the antifungal activity of TcPR-4b on *M. perniciosa*, increasing concentrations of the recombinant protein (0 to 40 μg/ml) were incubated with dikaryotic broken hyphae of *M. perniciosa*. The reduction of *M. perniciosa* survival was observed in all tested concentrations of TcPR-4b with a decrease of survival correlated to the increase of the protein concentration (Figure 11). In presence of 5 μg/ml of TcPR-4b the fungus survival did not present any difference in comparison to control (PBS without protein). At 10, 20 and 40 μg/ml of TcPR-4b, the survival decreased significantly and reached 10.3% in presence of 40 μg/ml of protein. The ROS production in living *M. perniciosa* hyphae treated with TcPR-4b was evaluated by incubation with DHE which selectively stains the mitochondrial superoxide (O_2_^-^). The hyphae treated with TcPR-4b presented a bright red fluorescence (Figure 11C and E) with more specific intense fluorescence in some foci (Figure 11E, arrows). The control (hyphae incubated with PBS) presented few or no fluorescence in comparison to the hyphae treated with TcPR-4b (Figure 11B and D). The action of TcPR-4b (40 μg/ml) on *M. perniciosa* survival was tested in presence (or not) of RNase inhibitor (Figure 12A). In presence of TcPR-4b plus RNase inhibitor the survival was high (83%) and statistically different from the survival in presence of TcPR-4b only (6%). The *M. perniciosa* survival in presence of TcPR-4b plus RNase inhibitor was similar as the fungus survival in presence of the controls (PBS and PBS plus RNase inhibitor). The *M. perniciosa* survival in presence of TcPR-4b with or without MgCl_2_ was similar and low (about 10%; Figure 12B).

## Discussion

In this article, we characterized a pathogenesis-related protein 4b from *Theobroma cacao* (TcPR-4b; Figure 1) previously identified in cacao-*M. perniciosa* interaction cDNA libraries. The TcPR-4b protein presented a Barwin domain – highly conserved among the PR-4s – but no chitin-binding domain (Figure 2). In comparison to the other six PR-4s from cacao, the TcPR-4b contains only one Barwin domain and no vacuolar signal (Figure 4). But the sequence analysis revealed that TcPR-4b contains a signal peptide, suggesting that it is an extracellular protein targeted to the apoplasm (Figures 2 and 4). TcPR-4b showed high identity with class II PR-4s from other plant species (Figure 2; Table 1).

The enzymatic tests carried out with the recombinant TcPR-4b revealed that this protein presented both DNase and RNase activities (Figures 8 and 9). To our knowledge, this is the second report of PR-4 with these two functions (Table 1). The TcPR-4b DNase activity is dependent of bivalent ions, Mg^2+^ and Ca^2+^ since chelation or absence of these ions prevent any DNase activity. In some studies, such as in the case of *Capsicum chinense* class II PR-4 (CcPR-4), the DNase catalytic function is independent of Mg^2+^, but addition of these ions reinforced the DNase activity [35]. On the contrary, the RNase activity of TcPR-4b was independent of bivalent ions, suggesting probable different catalytic sites for the two nuclease actions. As observed in other works [18,26,38], the RNase activity of TcPR-4b was inhibited by heating and in the presence of RNase inhibitor (RiboLock, Thermo Scientific) which is able to annul the activity of type A, B and C RNases. However, some exceptions were reported, for example, the CcPR-4 seemed to have a RNase activity mechanism different from those of the type A, B and C RNases [35]. Moreover, for class I PR-4 s, as FaPR4, the presence of the chitin-binding domain may contribute to the thermal stability of the protein avoiding the lack of RNase activity when the protein was submitted to heating [33].

The antifungal activity of TcPR-4b was verified *in vitro* on *M. perniciosa* hyphae (Figure 11A) and was associated to the increase of mitochondrial O_2_^-^ production detected by DHE (Figure 11C and E). The high production of O_2_^-^ should have altered the mitochondrial complex leading to oxidative stress, damage of DNA, lack of cellular functions, apoptosis or necrosis, and consequently inhibition of fungal growth. The action mechanism of the PR-4 probably involved the Arg_7_, His_11_, Asp_92_, His_113_ residues and the 15-PAQNNWD-21 region that are considered crucial for RNase and antifungal activities [18,39,52]. The structural model of TcPR-4b showed that the His_31_, His_130_, Arg_27_ residues and the region 35-PEQNNWD-41 are present in accessible regions of the protein; only the Asp_110_ amino acid had a more internal localization (Figure 3). Because of the accessibility of most of these residues in a highly flexible molecular structure, they may correspond, in TcPR-4b, to the interaction points with target molecules from *M. perniciosa*, and thus may be considered as good candidate for directed-site mutation studies.

The antifungal activity is common in different PR families, regardless of the specific enzymatic function of each of them [6,8,11,15]. In the case of the PR-4 family, the presence or absence of chitin-binding domain is not correlated with the antifungal action; class I [32,33,37] as well as class II [26,29,38,39] PR-4 proteins presented antifungal activity (Table 1). Moreover, the presence of this domain is not associated to the chitinolytic function of the PR-4 proteins (Table 1) as observed in the case of AtHEL [32] and FaPR-4C [33]. The FaPR-4C protein, a truncated form of a class I protein obtained to mimic a class II PR-4, presented the same chitinases, RNase and antifungal activity as the original non truncated FaPR-4 [33]. Moreover, the chitinase function was demonstrated for a very few number of PR-4s (Table 1); here the TcPR-4b neither presented chitin-binding domain nor chitinase activity (Figures 2, 4 and 10), but presented antifungal action against *M. perniciosa* (Figure 11). These data indicate that the classification of PR-4 as chitinases is inadequate and could be questioned, as previously suggested [39]. The doubts about the PR-4 protein function in relation to the presence/absence of the chitin-binding domain raises questions about the evolutionary origin of this domain. There is no consensus as to whether the chitin-binding domain was incorporated in class II proteins that contained a unique Barwin domain, or if the chitin-binding domain was present in all sequences and was lost later on. Phylogenetic analysis suggested that the class I genes evolved after the incorporation of the N-terminal domain in class II genes [53]. But other authors highlighted the hypothesis that the class II derived from class I after the lack of the hevein domain [30]. Here, the formation of a single clade only with most related PR4 sequences of *T cacao*, with duplicate Barwin domain and vacuolar signal, indicates a possible single origin for these molecules (Figure 5). Nevertheless, we find other *T cacao* PR4 grouped in class I, along with sequences of other fungal species. This last fact may indicate two evolutionary situations: PR4 class II emerged from a common class I ancestral, which lost the ability chitin binding, or a class II ancestral gave rise to two proteins of class I, Tc00 g012980 Tc05 g027320, which acquired the ability to bind to chitin. Considering that the interaction between *M. perniciosa* and *T. cacao*, during the various stages of growth of this fungus, is intense and evolutionarily ancient, it is possible that class II molecules in *T cacao* have given rise to class I.

Moreover, we showed that the antifungal activity of TcPR-4b is directly dependent of its RNase activity; the survival of *M. perniciosa* colonies was higher in presence of TcPR-4b plus RNase inhibitor than in presence of TcPR-4b only (Figure 12A). Experiment involving the TcPR-4b with or without MgCl_2_ (which is necessary for TcPR-4b DNase activity) did not show any difference, suggesting that the antifungal activity does not depend of the DNase activity of the protein (Figure 12B). The association between the antifungal and RNase activity has been proposed for the PR-4 family. Directed site-mutations of the wheatwin 1 protein (belonging to class II) on the conserved amino acids H_11_ and H_113_ (H_11_G, H_11_L ou H_11_L/H_113_L) considered as crucial for RNase activity, reduced the wheatwin 1 capacity to degrade RNA; in the double mutant H_11_L/H_113_L the reduction was higher than in the simple mutants [18]. The absence of RNase actitivy due to the mutation insertion was correlated with the reduction of antifungal potential [18]. The MdPR-4 protein also presented a correlated reduction of the RNase and the antifungal activities after treatment with a RNase inhibitor (5′-ADP) [26]. These evidences suggested that the antifungal activity depends on the RNase activity. Moreover, in the case of the wheatwin 1 protein, it has been shown that the protein is able to enter to the cytoplasm of the fungal cell and act as RNase without cell membrane destruction [18].

PR proteins are known to be involved in plant responses to infection by pathogens, and some PR-4 are more specifically associated to responses to fungal pathogens [2,23,26]. Here, we analyzed the expression of the *TcPR-4b* gene in resistant and susceptible cacao genotypes infected or not with *M. perniciosa.* At the early stages of the infection, the global *TcPR-4b* expression pattern was similar between resistant and susceptible infected plants even if a significant difference was observed at 48 hai between the two genotypes (Figure 6). This early expression may be considered as the first plant reaction against the pathogen in both genotypes, working as an induced defense mechanism against *M. perniciosa* basidiospore germination, and hyphae penetration and progression. The apoplastic addressing of the TcPR-4b (Figures 1 and 2), together with its nuclease activities (Figures 8 and 9), suggest that these two functions may be related to the antifungal activity of the protein (Figure 11) mainly during the biotrophic phase of the disease, when *M. perniciosa* grown intercellularly [54]. The higher intensity of the *TcPR-4b* expression in the resistant TSH1188 plants (2 times more than in susceptible ones; Figure 6) may contribute to the reduction of *M. perniciosa* hyphae penetration and progression in this genotype [54]. In susceptible cacao meristems, *M. perniciosa* hyphae were detected in the intercellular space of the sub-epidermis layer 24 hai while in the resistant genotype no or few fungal penetration was observed [54,55]. For most of the pathosystems, the response of plant resistance or susceptibility is more associated to differences of time/velocity and magnitude/intensity of some gene expression than to difference in gene set composition [3]. For example, during the Arabidopsis-*Colletotrichum higginsianum* interaction, the PR-4 expression was detected in both resistant and susceptible inoculated plants. In both genotypes, the expression increased through the time course of the disease, but the highest PR-4 expression was observed at 48 hai in the resistant genotype and only 4 dai in the susceptible one [56]. This result was in accordance with literature data showing that, generally, a higher PR protein accumulation and/or an earlier gene expression was observed in the incompatible interaction rather than in the compatible one [6,24,35,57,58]. After the initial stage, in the resistant cacao plants, the *TcPR-4b* expression was observed in all the samples with a relatively high level (from 2 to 8 times higher than the control) while a gene repression was observed from 8 to 30 dai in susceptible plants (compared to the control) followed by an increase of expression from 45 to 90 dai (Figure 6). In the resistant cacao genotype, even if no macroscopic symptom were observed, some metabolic alterations have been related after inoculation of the plants by *M. perniciosa*[45,54,59]. Among them, H_2_O_2_ production associated to calcium oxalate crystal (COC) dissolution as well as expression of genes involved in Ca^2+^ dependent-signalization were observed at the initial stages after inoculation (24, 48 and 72 hai) [45,54,59]. Moreover, a germin-like oxalate oxidase – responsible for COC dissolution – was expressed constantly in the resistant cacao genotype, making a high Ca^2+^ amount available a long time after the plant has been in contact with the pathogen [54,59]. In the susceptible plants, COCs were accumulated in cortex during the biotrophic phase (until 30 dai; [54,60]) and dissolved during the transition to the necrotrophic phase (from 30 to 50 dai) [54]. Moreover, the necrophic phase is characterized in the susceptible plant by a nuclear DNA degradation associated to programmed cell death (PCD) [54]. Considering that the TcPR-4b DNase activity is Ca^2+^-dependent, the high and almost constant expression of the corresponding gene in the TSH1188 genotype from 30 to 90 dai, could reflect the role of TcPR-4b in a protection mechanism against further foreign invading pathogens, as suggested for PR-4 from other plants [35]. In the Catongo plants, the *TcPR-4b* expression coincided with the COC dissolution during biotrophic/necrotrophic phase transition as well as to the nuclear DNA degradation and ladder formation during the necrotrophic phase [54] suggesting that in this genotype, the TcPR-4b may act as one of the element of the PCD process. The increase of nuclease activity, some of them Ca^2+^ and Mg^2+^ dependent have been identified in plants during PCD processes [61-65]. For example, in wheat seeds the highest accumulation of PR-4 occurred in period concomitant with PCD of the endosperm [66]. In conclusion, in cacao, the COC dissolution which occurred in both resistant and susceptible genotypes led to extracellular Ca^2+^ availability that may be used by the TcPR-4b for its acting as DNase in resistance mechanisms or in PCD during the infection process. This may be reinforced by the fact that the calcium availability is higher in the apoplasm in normal cells, while an influx of calcium in the intracellular area occurred during PCD [63].

## Conclusion

We reported the first evidence of a PR-4 with a dual DNase and RNase activity together with an antifungal activity. To our knowledge, this is the first report of PR-4 showing these three functions simultaneously, but no chitinase activity (Table 1). Moreover, we showed that the antifungal activity of TcPR-4b is directly related to the RNase function – and not to the DNase one (Figure 12). These functions could be related to the role of the TcPR-4 during the cacao-*M. perniciosa* interaction, contributing to the maintenance of the symptom reduction in the resistant genotype and to the PCD mechanism in susceptible one. Thus, the TcPR-4b could be considered as a good candidate for biotechnological applications in order to detain witches’ broom disease.

## Methods

### Sequence analysis

The *TcPR-4b* cDNA was identified from a library of *Theobroma cacao* L. pod (genotype TSH1188) infected by *Moniliophthora perniciosa* (accession number FC072496.1), and was also present in cDNA library from cacao meristem infected by *M. perniciosa* (Gesteira et al., 2007; accession number ES440503.1). Open reading frame (ORF) analysis was performed using the ORFinder software. Sequence homology search was made with BLAST [67] on the National Center for Biotechnology Information (NCBI) and CocoaGenDB [47] databases. Multiple sequence alignment was performed with the ClustalW2 software [68]. The prediction of theoretical isoelectric point (pI) and molecular weight (MW) were obtained using the Expasy Molecular Biology Server (http://www.expasy.org). The conserved domain and family protein were analyzed using the Pfam (http://pfam.sanger.ac.uk/search/sequence) and InterProScan [69] programs. NetPhos 2.0 Server [70] and NetNGlyc 1.0 Server (http://www.cbs.dtu.dk/services/NetNGlyc/) were used for identification of putative phosphorylation sites (Ser/Thr/Tyr) and putative N-glycolsylation sites (Asn-X-Ser/Thr type), respectively. The ASEB server was used to predict putative KAT-specific acetylation sites [71]. Signal peptide presence was analyzed using SignalP 4.0 Server [48]. Transmembrane helices were predicted by TMHMMA server 2.0 (http://www.cbs.dtu.dk/services/TMHMM/; [72]).

### Phylogeny

Phylogenetic analysis was performed for all amino acid sequences from all *T. cacao* and related to TcPR-4b sequence. The sequences were aligned by ClustalW2 (http://www.ebi.ac.uk/Tools/msa/clustalw2/) [68], and choosing BLOSUM matrix [73]. The alignment was saved in NEXUS format for the phylogenetic analysis. Then, a Bayesian calculation with MRBAYES 3.1.2 [74], using the mixed evolutionary model with three independent runs (each with four chains) for 1 × 10^6^ generations, and sampling every 100 generations, was performed. For this analysis the *Oryza sativa* sequence (OsPR-4b) was used as an outgroup, because of its more distant evolution relationship with *T. cacao*.

### Molecular modeling

The prediction of the three-dimensional (3-D) models of the TcPR-4b protein was obtained using the Swiss Pdb-Viewer software v.3.7 [75]. To select the best 3-D template to be used for molecular modeling of TcPR-4b from resolved 3-D structures, the TcPR-4b amino acid sequence was subjected to alignment using the PSI-BLAST program against the Protein Data BankProtein (pdb) [76]. The best alignment (E-value 3.10^-62^) was obtained with the barley Barwin protein (PDB code 1BW3_A.pdb) which presents a single structure resolved in solution by nuclear magnetic resonance spectroscopy (NMR Resolution: 99.9; [34]); the barley barwin protein was thus used to build the TcPR-4b 3-D model. The root mean square deviation (RMSD) differences from ideal geometries of bond lengths and bond angles were calculated on PyMOL V3.0 (The PyMOL Molecular Graphics System, Schrödinger, LLC). The stereochemical quality of the TcPR-4b 3-D model was assessed using the Procheck 3.4 [77] and the ANOLEA (Atomic Non-Local Environment Assessment; [78]) programs. The validation of the secondary structure was performed using the program PSIPRED (Protein Structure Prediction Server; http://bioinf.cs.ucl.ac.uk/psipred/; [79]).

### Plant material

Seeds of *Theobroma cacao* L. genotypes Catongo (susceptible to *M. perniciosa*) and TSH1188 (resistant to *M. perniciosa*) were germinated and grown at CEPLAC/CEPEC (Bahia, Brazil) greenhouses. Twenty to thirty days after germination, the apical meristems of the plantlets were inoculated by the droplet method [80] with a basidiospore suspension (2.10^5^ basidiospore.ml^-1^) of *M. perniciosa* (inoculum from isolate 4145 maintained in the CEPLAC/CEPEC phytopathological *M. perniciosa* collection under number 921 of the WFCC; http://www.wfcc.info/index.php/collections/display) [80]. After inoculation, the plantlets were kept for 24 h at 25 ± 2°C and 100% humidity. Rate of disease fixation based on presence/absence of symptoms [81] in each genotype, was evaluated 60 days after inoculation (dai); disease rate was 45% and 80% for TSH1188 and Catongo, respectively. Moreover, the presence of *M. perniciosa* in the plant material was checked by semi-quantitative RT-PCR using specific *M. perniciosa* actin primers [82]; both genotypes presented fungus incidence (data not shown) coherent with previous data obtained in the same conditions of plant culture and inoculation [55]. Apical meristems were harvested at 24, 48 and 72 hours after inoculation (hai), and 8, 15, 30, 45, 60 and 90 dai. Non-inoculated plants (controls) were kept and harvested under the same conditions at 24 and 72 hai, and 30, 60 dai and 90 dai. For each genotype and at each harvesting time (for inoculated and non inoculated plants), 20 samples were collected (1 sample = 1 apical meristem of 1 cacao plantlet). The 20 samples collected from one genotype at one harvesting time were pooled; thus 9 inoculated and 5 non inoculated (control) samples were immediately frozen in liquid nitrogen and stored at -80°C until use. Pooling samples before RNA extraction has the advantage of reducing the variation caused by biological replication and sample handling [83].

### Reverse transcription quantitative PCR analysis

Cacao samples were macerated in liquid nitrogen until obtaining a fine powder. Total RNA was extracted from macerated samples using the RNAqueous Kit® (Ambion) according to the manufacturer’s instructions, with modifications. After the addition of the lysis buffer to the macerated samples, a sonication step was added (10 s pulse/min, 70% output; Gex Ultrasonic processor 130, 130 W) to break polysaccharides which are present in high levels in cacao tissues [84]; this step was conducted on ice. The synthesis of the first cDNA strand was carried out using Revertaid Fisrt Strand cDNA Synthesis Kit according to the manufacturer’s instructions (Thermo Scientific). The cDNA quantification was carried out on the GeneQuant pro UV/Vis spectrophotometer (Amersham). For the qPCR analysis, the expression of three cacao endogenous reference genes, the malate dehydrogenase (MDH), glyceraldehyde 3-phosphate dehydrogenase (GAPDH) and β-actin (ACT), previously identified as *T. cacao* housekeeping genes [85], was analyzed on cacao meristems using the NormFinder program [86]. Specific primers and amplified regions containing different size, melting temperature, GC content and GC/AT ratio were defined to avoid cross-reaction between genes from cacao PR4 family [87]. Expression analysis by qPCR was performed using standard settings of the ABI PRISM 7500 and Sequence Detection System (SDS) software, version 1.6.3 (Applied Biosystems). The qPCR reaction consisted of 10 ng/μl of cDNA, 200 μM of each primer from reference or *TcPR-4b* genes (Table 2) and 11 μl of Power SYBR Green Master Mix (Applied Biosystems) in a total volume of 25 μl. Cycling conditions were: 50°C for 2 min then 95°C for 10 min, followed by 40 cycles at 95°C for 15 s, 60°C for 30s and 60°C for 1 min. To verify that each primer pair produced only a single PCR product, a dissociation analysis was carried out from 60°C to 95°C and analyzed with the Dissociation Curve 1.0 program (Applied Biosystems). The gene expression level was analyzed on six experimental repetitions for both Catongo and TSH1188 genotypes with the comparative Ct method (2^-ΔΔCt^) using: i) MDH and ACT as reference genes (average of expression values from both genes); and ii) non-inoculated plants (average of expression values of *TcPR-4b* in 5 control samples harvested as described above) as calibrator - for this reason the relative expression value of the control (=non-inoculated plants) is always 1.0. Statistical analysis was made using the SASM-Agri software [88] which tested the experiments as a completely randomized design. *t*-test and *F*-test (ANOVA) were applied with a critical value of 0.01. The Scott-Knott (P ≤ 0.01) test was employed for mean separation when *F*-values were significant.

**Table 2:** Primers used for qPCR analysis

**Name**	**Sequence**	**Size of the amplified product (bp)**	**Tm (°C)**	**Reference**
ACT-F	5′- TCCTCTTCCAGCCATCTCTC-3′	171	56	[85]
ACT-R	5′- TCTCCTTGCTCATTCGGTCT-3′		56	
MDH-F	5′- AAAATGGAGTTGGTGGATGC-3′	102	54	
MDH-R	5′- AACCATGACTGCGATGTTGA-3′		55	
GAPDH-F	5′- GATGCTCCTATGTTTGTTGTGG-3′	222	54	
GAPDH-R	5′- TCTTCCTCCTCTCCAGTCCTT-3′		57	
TcPR-4b-F	5′- TGACCGCTGTAAGTGCTTTCTG-3′	81	57	This study
TcPR-4b-R	5′-AGGCCGTCCATCCATATTTG-3′		55	

### Expression of recombinant TcPR-4b

The *TcPR-4b* cDNA was amplified by PCR from the *pDNR-LIB::TcPR-4b* construction using 200 μM of TcPR-4bF (5′-GCGGCATATGCAAAGCGCTTCCAATGTG-3′/*Nde*I underlined site) and TcPR-4bR (5′-GCGGGGATCCTTAGTCACCACAATC-3′/*Bam*HI underlined site) primers, 1.5 mM of MgCl_2_, 0.25 μM of dNTPs, 1X Taq buffer containing (NH_4_)_2_SO_4_, 1U of Taq polymerase (Thermo Scientific), under the following conditions: 94°C for 4 min, followed by 35 cycles at 94°C for 30s, 52°C for 45 s and 72°C for 1 min. The PCR product was cloned into the *Nde*I and *Bam*HI sites of the pET28a plasmid (Novagen) using the T4 DNA ligase (Thermo Scientific), and the resulting in frame fusion plasmid was used to transformed *Escherichia coli* Roseta (DE3) for protein expression. This system allows the production of a recombinant His-Tag protein. To establish an efficient strategy of TcPR-4b production, the induction was carried out at two temperatures (18 and 37°C), two isopropyl-β-D-thio-galactoside (IPTG) concentrations (0.4 and 1 mM) and different induction periods (16 h for induction at 18°C, and 1, 2, 3 and 4 h for induction at 37°C). After induction, the cells were collected by centrifugation (11.000 *g*, 20 min, 4°C) and the pellets ressuspended in lysis buffer (6 M urea, 50 mM phosphate buffer, 300 mM NaCl, 2% Nonidet, 0.1 mg.ml^-1^ lysozyme, pH 8). The purification of the recombinant N-terminal histidine-tagged TcPR-4b was performed by immobilized metal affinity chromatography (IMAC) with TALON® Metal Affinity Resin according to the manufacturer’s instructions (Clontech). The TcPR-4b protein, which was expressed only in the insoluble fraction, was dialyzed using the HisTrap™ FF crude (GE Healthcare) to remove urea. The presence of the TcPR-4b protein after expression, purification and dialysis was analyzed on 15% sodium dodecyl sulfate polyacrylamide gel electrophoresis (SDS-PAGE) as previously described [89]. Electrophoresis ran for 2 h at 150 V with the cube immersed in ice, and the proteins were detected on electrophoresis gel by Coomassie Blue G 250 staining [90]. Protein concentration was analyzed in Qubit® 2.0 Fluorometer using Qubit® protein assay kit (Invitrogen).

### Ribonuclease and deoxyribonuclease activity of recombinant TcPR-4b

RNase activity of the recombinant purified TcPR-4b was performed using different protein concentrations (5, 10, 15, 20 and 25 μg) incubated with 5 μg of extracted RNA from tomato (*Solanum lycopersicum* var. Micro-Tom) leaves using the RNAqueous Kit® according to the manufacturer’s instructions (Ambion). The reaction was incubated at 25°C for 30 min and the product was analyzed in 1.5% agarose electrophoresis gel. Bovine serum albumin (25 μg; BSA, Sigma) and RNase A (25 μg; Thermo Scientific) were used as negative and positive controls, respectively. Inhibition of the RNase activity was evaluated using the RiboLock RNase Inhibitor (40 U/μl; Thermo Scientific). DNAse activity of the recombinant purified TcPR-4b protein was tested in the presence or absence of bivalent calcium (Ca^+2^) and magnesium (Mg^+2^) ions. The assays were performed with 1 μg of purified plasmidial DNA (pGEM-T® Easy Vector; Promega) incubated with different TcPR-4b amounts (2, 4, 6, 8, 10, 12, 14, 16, 18 and 20 μg) in the presence or absence of 10 mM of MgCl_2_ (from Taq DNA Polymerase kit, Thermo Scientific) overnight at 25°C. The same procedure was done incubating the plasmidial DNA with 5, 10, 15 and 20 μg of the TcPR-4b protein in the presence of 1 mM of CaCl_2_ (higher concentrations led to protein precipitation). To inhibit the DNase activity, 10 mM of the chelating agent ethylenediamine tetraacetic acid (EDTA) were added to the reaction containing 10 mM of the MgCl_2_ and 10 or 20 μg of TcPR-4b. DNase activity was also tested in genomic DNA (gDNA) by incubation of 6 μg of TcPR-4b with 1 μg of gDNA from *Nicotiana tabacum* in the presence or absence of 10 mM de MgCl_2_ overnight at 25°C.

### Antifungal activity of recombinant TcPR-4b

The recombinant purified TcPR-4b was used for *in vitro* antifungal activity assays against dikaryotic *M. perniciosa* (mycelium from isolate 4145 maintained in the CEPLAC/CEPEC phytopathological *M. perniciosa* collection under the number 921 of the WFCC) broken hyphae. One centimeter disc plugs from a 15-days-old mycelium culture grown on solid mineral medium (0.1% NH_4_H_2_PO_4_ [w/v], 0.02% KCl [w/v], 0.02% MgSO_4_.7H_2_O [w/v], 0.001% CuSO_4_.5H_2_O [w/v], 0.001% ZnSO_4_.7H_2_O [w/v], 0.5% yeast extract [w/v] and 1.5% agar [w/v]) were broken with glass beads (Sigma, G1277) under vigorous vortexing for 60 s and grown for 7 days in CPD liquid medium (2% glucose, 2% peptone) at 25°C as previously described [91]. Afterwards, 1 ml of the broken hyphae suspension was incubated for 2 h with: i) 5, 10, 20 or 40 μg of TcPR-4b in phosphate buffer saline (PBS); ii) only PBS (control). In order to evaluate the relation between fungal survival in presence of TcPR-4b and RNase activity, 1 ml of broken hyphae suspension was incubated for 2 h with: i) PBS (control); ii) PBS plus 800 U of Ribolock RNase inhibitor (Thermo Scientific); iii) 40 μg/ml of TcPR-4b; iv) 40 μg/ml of TcPR-4b plus 800 U of Ribolock RNase inhibitor. In order to evaluate the relation between fungal survival in presence of TcPR-4b and DNase activity, 1 ml of broken hyphae suspension was incubated for 2 h with: i) PBS (control); ii) PBS plus 10 mM MgCl_2_; iii) 40 μg/ml of TcPR-4b; iv) 40 μg/ml of TcPR-4b plus 10 mM MgCl_2_. Then, 1 ml of each treatment was applied on CPD solid medium (CPD liquid medium plus 2% of agar poured into the Petri plates) and incubated for 7 days at 25°C. After the incubation period, the survival was examined by counting the number of pseudo-colonies on three experimental replicates (three plates). Moreover, three independent experiments were carried out, totalizing nine repetitions used for statistical analyses. Data were subjected to the Shapiro Wilk normality test followed by the analysis of variance with Tukey test (α = 0.05). Analyses were carried out using the Bioestat v4.0 [92] and SAMS-AGRI [88] softwares.

### Reactive oxygen species detection

To detect the production of mitochondrial superoxide anion (O_2_^-^) in living cells of *M. perniciosa*, 1 ml of broken dikaryotic hyphae suspension (obtained as described above) was incubated with 10 μg of TcPR-4b in PBS overnight at 25°C. As control experiment, hyphae were incubated only with PBS. Then, the *M. perniciosa* hyphae pretreated with the TcPR-4b protein were incubated with 10 mM of dihydroethidium (DHE; Sigma) at 25°C for 30 min. The chemical reaction involved the DHE reaction with O_2_^-^, forming ethidium that intercalated with DNA and then emitted red fluorescence [93-95]. The hyphae were mounted on slides and observed under fluorescence microscope BX51 (Olympus). Images were captured using × 40 and × 100 objectives under fluorescent filters using the Image Pro software v.6.3. (Olympus).

### Chitinase activity

Chitinase activity was determined by colorimetric assays using the purple dye-labeled biopolymeric substrate, CM-chitin-RBV (Loewe Biochemical, Germany). As positive control, cacao meristems infected by *M. perniciosa* and harvested in the field the Universidade Estadual de Santa Cruz (Ilhéus, Bahia, Brazil), were used [96]. Cacao meristems were ground in liquid nitrogen and 1 g of the macerated tissue was used for protein extraction using 5 ml of extraction buffer A (10 mM of sodium acetate pH 5.0, 5 mM of phenylmethylsulfonyl fluoride-PMSF, 1% of polyvinylpyrrolidone-PVP) or extraction buffer B (10 mM Tris-HCl pH 7.0, 5 mM of PMSF, 1% of PVP). Samples were sonicated (10 s pulse/min, 70% output; Gex Ultrasonic processor 130, 130 W) and centrifuged at 20,000 *g* for 20 min at 4°C; the supernatant was collected. The recombinant TcPR-4b purified in 50 mM of PBS (pH 8.0) was dialyzed in buffer A or buffer B using the Spectra/Por® Dialysis membrane. Protein concentration of total cacao extract and TcPR-4b was measured on Qubit® 2.0 Fluorometer using the Qubit® protein assay kit according to the manufacturer recommendations (Invitrogen). The reactions containing 200 μl of protein extract (cacao or TcPR-4b) at different concentrations (20, 40, 80, 100 μg), 400 μL sodium acetate buffer (10 mM, pH 5.0) or Tris-HCl buffer (10 mM, pH 7.0) and 200 μL of CM-chitin-RBV, were incubated at 40°C for 2 h. The reaction was stopped by the addition of 200 mL of 2 M HCl. Samples were cooled on ice for 10 min, then centrifuged at 20,000 *g* for 10 min at 4°C to remove the non degraded substrate. The supernatant was collected and the assay was performed spectrophotometrically at 550 nm (SpectraMax Paradigm, Multi-Mode Detectiar Plataform) using the SoftMax Pro v.6.3. Chitinase activity was described by unit/h. One unit of chitinase activity corresponded to an increase of absorbance of 0.1 [97]. For each sample, four independent replicates were used.

## Availability of supporting data

The data sets supporting the results of this article are included within the article and its additional files.

## Abbreviations

ABA: Abscisic acid; ACT: β-actin; BSA: Bovine serum albumin; dai: Day after inoculation; COC: Calcium oxalate crystal; DHE: Dihydroethidium; ET: Ethylene; GAPDH: Glyceraldehyde 3-phosphate dehydrogenase; hai: Hour after inoculation; IPTG: Isopropyl-β-D-thio-galactoside; JA: Jasmonate; MDH: Malate dehydrogenase; MW: Molecular weight; ORF: Open reading frame; pI: Isoelectric point; PBS: Phosphate buffered saline; PCD: Programmed cell death; RMSD: Root mean square deviation; ROS: Reactive oxygen species; SA: Salicylic acid.

## Competing interest

No conflicts of interest to declare.

## Authors’ contributions

SPM and FM were responsible for the conception and design of the experiments, the analysis of the data and the redaction of the manuscript; SPM was responsible for bioinformatics (except phylogeny) and for the execution of all the experiments; EMAS participated of the TcPR4 production and purification; EML participated to the RNA extraction and cDNA production; AOS helped in qPCR experiment design and analysis; BSA developed the phylogeny analysis; LSLL obtained and kindly gave the *TcPR-4b* cDNA clone; KPG was responsible for plant material production and inoculation with *M. perniciosa*; ASG helped in *TcPR-4b* cloning; CPP helped in all biochemical steps of the work and availed the laboratorial infrastructure; FM was responsible for the financial support of the research and for the advising of SPM, EMAS and EML. All authors read and approved the final manuscript.

## Supplementary Material

Additional file 1 **Complete *TcPR4-b *** **gene sequence (802 bp) obtained from CocoaGenDB.** The 5’UTR (37 bp) is underlined. The exons (171 bp and 258 bp) are highlighted in black. The intron (82 bp) is indicated in gray. The 3’UTR (254 bp) is underlined and in italic. Click here for file

Additional file 2 **Pathogenesis-related proteins 4 from *Theobroma cacao *** **obtained by BlastX on CocoaGenDB databank.** aa: amino acids. Click here for file

Additional file 3 **Validation of the built model of TcPR-4b.** Ramachandran plot obtained with PROCHECK software. Red, yellow, light yellow and white regions represent energetically most favored, allowed, generously allowed and disallowed regions, respectively. Click here for file

Additional file 4 **Dissociation curves of ACT, MDH and TcPR-4b in Catongo (A, C, E) and TSH1188 (B, D, F), respectively.** For all the harvesting point, the PCR amplification occurred at the same melting temperature showing that only the *TcPR-4b* gene was amplified. Click here for file

Additional file 5 **RNase and DNase activity test using the extract of the bacteria containing the pET28 vector without insert (negative control), avoiding the possible action of some bacterial component on the obtained result.** A. DNase activity. B. RNase activity. Click here for file
