# Supplementary material for: The pathogenesis-related protein PR-4b from Theobroma cacao presents RNase activity, Ca2+ and Mg2+ dependent-DNase activity and antifungal action on Moniliophthora perniciosa
Source: BMC Plant Biol. 2014 Jun 11;14:161. doi: 10.1186/1471-2229-14-161 (PMC4079191; doi:10.1186/1471-2229-14-161)
Supplement: Additional file 1 — Complete TcPR4-b gene sequence (802 bp) obtained from CocoaGenDB. The 5’UTR (37 bp) is underlined. The exons (171 bp and 258 bp) are highlighted in black. The intron (82 bp) is indicated in gray. The 3’UTR (254 bp) is underlined and in italic. [file 1471-2229-14-161-S1.docx]

**Additional file 1.** Complete *TcPR4-b* gene sequence (802 bp) obtained from CocoaGenDB. The 5’UTR (37 bp) is underlined. The exons (171 bp and 258 bp) are highlighted in black. The intron (82 bp) is indicated in gray. The 3’UTR (254 bp) is underlined and in italic.

1 ttttaggcaacaaaaactgcagtaaaagggggggaaaatgaaaatggagagattctgcat 60

61 tctgttgttagcttgtctggtggcttctgctgctgcccaaagcgcttccaatgtgagagc 120

121 tacttaccatttatataaccctgagcagaataactgggacttgaccgctgtaagtgcttt 180

181 ctgcgccacctgggatgccaataagcctctggaatggcgccgcaaatatggatggacggc 240

241 cttttgtggtccagctggtcctcgagggcaagctgcttgcggcaggtgcttaagggtaag 300

301 caatttttagctttaactgcttaggccacaatagtagttaaatagcagcacctaaaagtt 360

361 tattcttgcttgtgtaggtcacgaacaccgggaccggagctcaggcaacggtgagaatcg 420

421 ttgatcagtgcagcaacggaggcctagatttggatgtcaatgtgtttagacaacttgaca 480

481 caaatgggaacggcattgcacaaggccacctaattgtgaactatgactttgtggattgtg 540

541 gtgactaa*aagccctttgccctgttctcccaagcaatttttaagttacaataagagtttc* 600

601 *cagtgcctcatgaataaattatctctaagaaaataaagttgctacttgtaaactctataa* 660

661 *tgatacatatatattgttgtgaagagaagggagatatgattatgaacaaatttttctact* 720

721 *atgcatcaggcatcagcttcctctggtctcaaagtagcatttcctgccatattgcaaagg* 780

781 *aaaacgttctttatgttcttac* 802
