# Supplementary material for: The pathogenesis-related protein PR-4b from Theobroma cacao presents RNase activity, Ca2+ and Mg2+ dependent-DNase activity and antifungal action on Moniliophthora perniciosa
Source: BMC Plant Biol. 2014 Jun 11;14:161. doi: 10.1186/1471-2229-14-161 (PMC4079191; doi:10.1186/1471-2229-14-161)
Supplement: Additional file 2 — Pathogenesis-related proteins 4 from Theobroma cacao obtained by BlastX on CocoaGenDB databank. aa: amino acids. [file 1471-2229-14-161-S2.docx]

**Additional file 2.** Pathogenesis-related proteins 4 from *Theobroma cacao* obtained by BlastX on CocoaGenDB databank. aa: amino acids.

|  |  |  |  |  |  |  |  | Chromosome localization | | Size | |  |  |
| --- | --- | --- | --- | --- | --- | --- | --- | --- | --- | --- | --- | --- | --- |
| Nº | CocoaGenDB identification | Function | Class | E-value | Identity with TcPR-4b (%) | Acession number NCBI | Chromosome | Start | End | ORF (bp) | Protein (aa) | Exon/intron | Phosphorylation sites |
| 1 | Tc05_g027210^a^ | Pathogenesis-related protein PR-4B | II | 9.10^-81^ | 100 | [EOY11055.1](http://www.ncbi.nlm.nih.gov/protein/508719158?report=genbank&log$=prottop&blast_rank=1&RID=1U9F6EX6015) | 5 | 23.010.771 | 23.011.572 | 802 | 142 | 2/1 | T_97_ |
| 2 | Tc00_t012980 | Pro-hevein | I | 1.10^-55^ | 78.69 | [EOY25834.1](http://www.ncbi.nlm.nih.gov/protein/508778578?report=genbank&log$=prottop&blast_rank=1&RID=23WYTP5T01R)^b^ | 0^d^ | 13.937.546 | 13.940.240 | 2695 | 193 | 1/0 | S_53_, S_55_, T_47_, T_139_, T_143_, Y_51_, Y_186_ |
| 3 | [Tc05_g027320](http://cocoagendb.cirad.fr/gbrowse/cgi-bin/gbrowse/theobroma?name=Tc05:23076272..23077162) | Pro-hevein | I | 6.10^-49^ | 68.50 | [EOY11066.1](http://www.ncbi.nlm.nih.gov/protein/508719169?report=genbank&log$=prottop&blast_rank=1&RID=23Y7EKVT01R) | 5 | 23.076.272 | 23.077.162 | 891 | 207 | 2/1 | S_67_, S_109_, S_155_, S_200_, S_205_, T_6_, T_33_, T_73_, T_171_, T_182_, Y_44_, Y_53_, Y_114_, Y_176_ |
| 4 | Tc05_027220 | Pathogenesis-related protein P2 | II | 5.10^-44^ | 60.28 | [EOY11058.1](http://www.ncbi.nlm.nih.gov/protein/508719161?report=genbank&log$=prottop&blast_rank=1&RID=23YCGGUN01R) | 5 | 23.015.298 | 23.017.340 | 2043 | 271 | 3/2 | S_5_, S_22_, S_162_, T_94_, T_212_, Y_33_, Y_92_, Y_156_, Y_184_ |
| 5 | Tc05_g027250 | Pathogenesis-related protein P2 | II | 2.10^-38^ | 55.32 | [EOY11059.1](http://www.ncbi.nlm.nih.gov/protein/508719162?report=genbank&log$=prottop&blast_rank=1&RID=23PYB7ZV01R) | 5 | 23.029.426 | 23.031.764 | 2.339 | 277 | 3/2 | S_5_, S_22_, T_94_, T_145_, T_218_, T_223_, Y_33_, Y_92_, Y_133_, Y_190_ |
| 6 | Tc10_g011130 | Pathogenesis-related protein P2 | II | 1.10^-37^ | 55.80 | [EOY19131.1](http://www.ncbi.nlm.nih.gov/protein/508727234?report=genbank&log$=prottop&blast_rank=1&RID=23SJSPKG01R) | 10 | 9.724.346 | 9.726.117 | 1772 | 272 | 3/2 | S_5_, S_22_, T_94_, T_130_, T_213_, Y_33_, Y_61_, Y_133_, Y_157_, Y_185_, Y_257_ |
| 7 | [Tc05_g027230](http://cocoagendb.cirad.fr/gbrowse/cgi-bin/gbrowse/theobroma?name=Tc05:23022195..23023859) | Pathogenesis-related protein P2 | II | 6.10^-37^ | 55.40 | EOY11057.1^c^ | 5 | 23.022.195 | 23.023.859 | 1665 | 304 | 3/2 | S_11_, S_37_, S_54_, T_30_, T_126_, T_245_, Y_65_, Y_93_, Y_124_, Y_165_, Y_189_, Y_217_ |

^a^ Corresponds to TcPR-4b

^b^ Showed 99% of identity with NCBI protein

^c^ Showed 91% of identity with NCBI protein

^d^ Without defined localization on one of the 10 chromosomes of *T. cacao*
