# Supplementary material for: The pathogenesis-related protein PR-4b from Theobroma cacao presents RNase activity, Ca2+ and Mg2+ dependent-DNase activity and antifungal action on Moniliophthora perniciosa
Source: BMC Plant Biol. 2014 Jun 11;14:161. doi: 10.1186/1471-2229-14-161 (PMC4079191; doi:10.1186/1471-2229-14-161)

**Additional file 3.** Validation of the built model of TcPR-4b. Ramachandran plot obtained with PROCHECK software. Red, yellow, light yellow and white regions represent energetically most favored, allowed, generously allowed and disallowed regions, respectively.


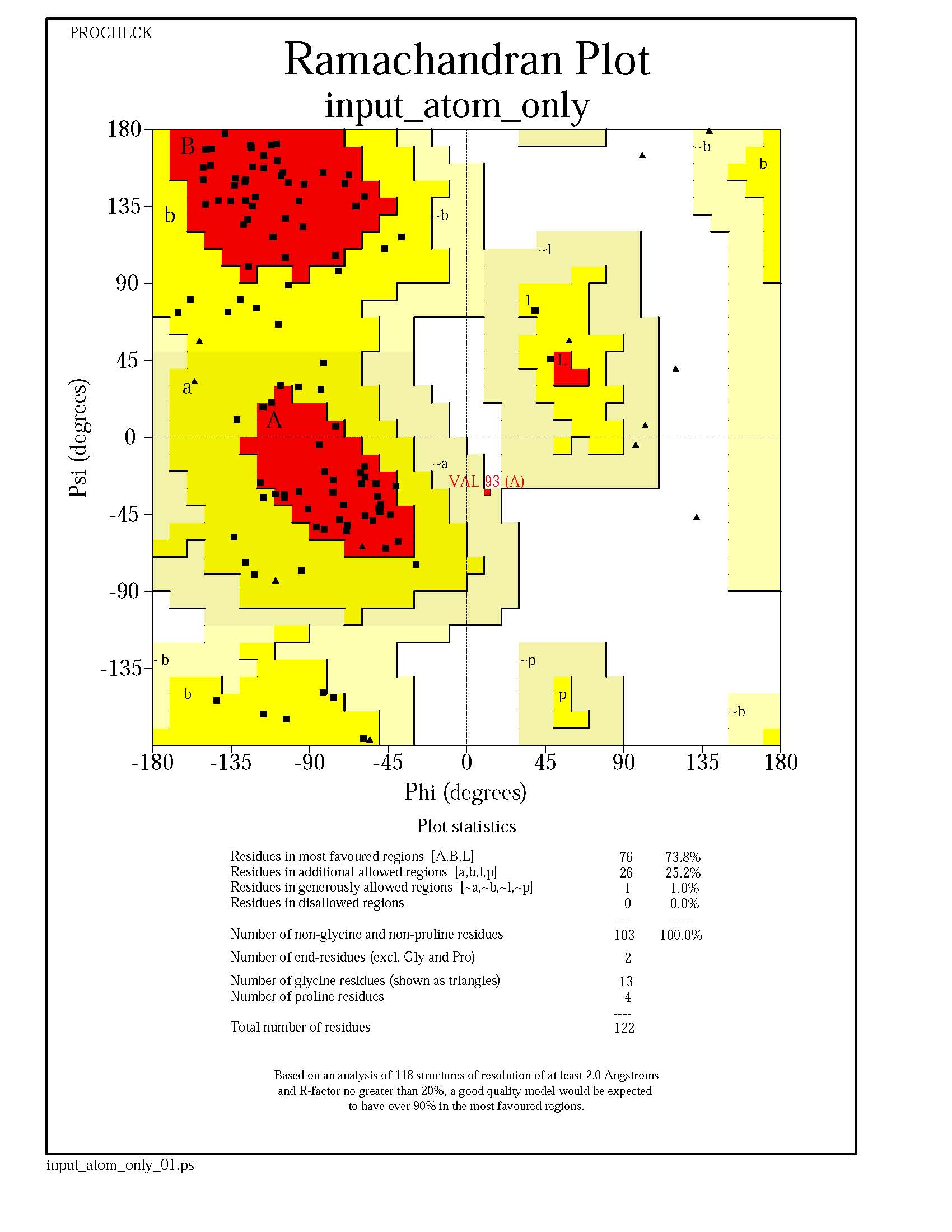

Supplement: Additional file 3 — Validation of the built model of TcPR-4b. Ramachandran plot obtained with PROCHECK software. Red, yellow, light yellow and white regions represent energetically most favored, allowed, generously allowed and disallowed regions, respectively. [file 1471-2229-14-161-S3.docx]
