# Supplementary material for: The pathogenesis-related protein PR-4b from Theobroma cacao presents RNase activity, Ca2+ and Mg2+ dependent-DNase activity and antifungal action on Moniliophthora perniciosa
Source: BMC Plant Biol. 2014 Jun 11;14:161. doi: 10.1186/1471-2229-14-161 (PMC4079191; doi:10.1186/1471-2229-14-161)

**Additional file 4.** Dissociation curves of ACT, MDH and TcPR-4b in Catongo (A, C, E) and TSH1188 (B, D, F), respectively. For all the harvesting point, the PCR amplification occurred at the same melting temperature showing that only the *TcPR-4b* gene was amplified.


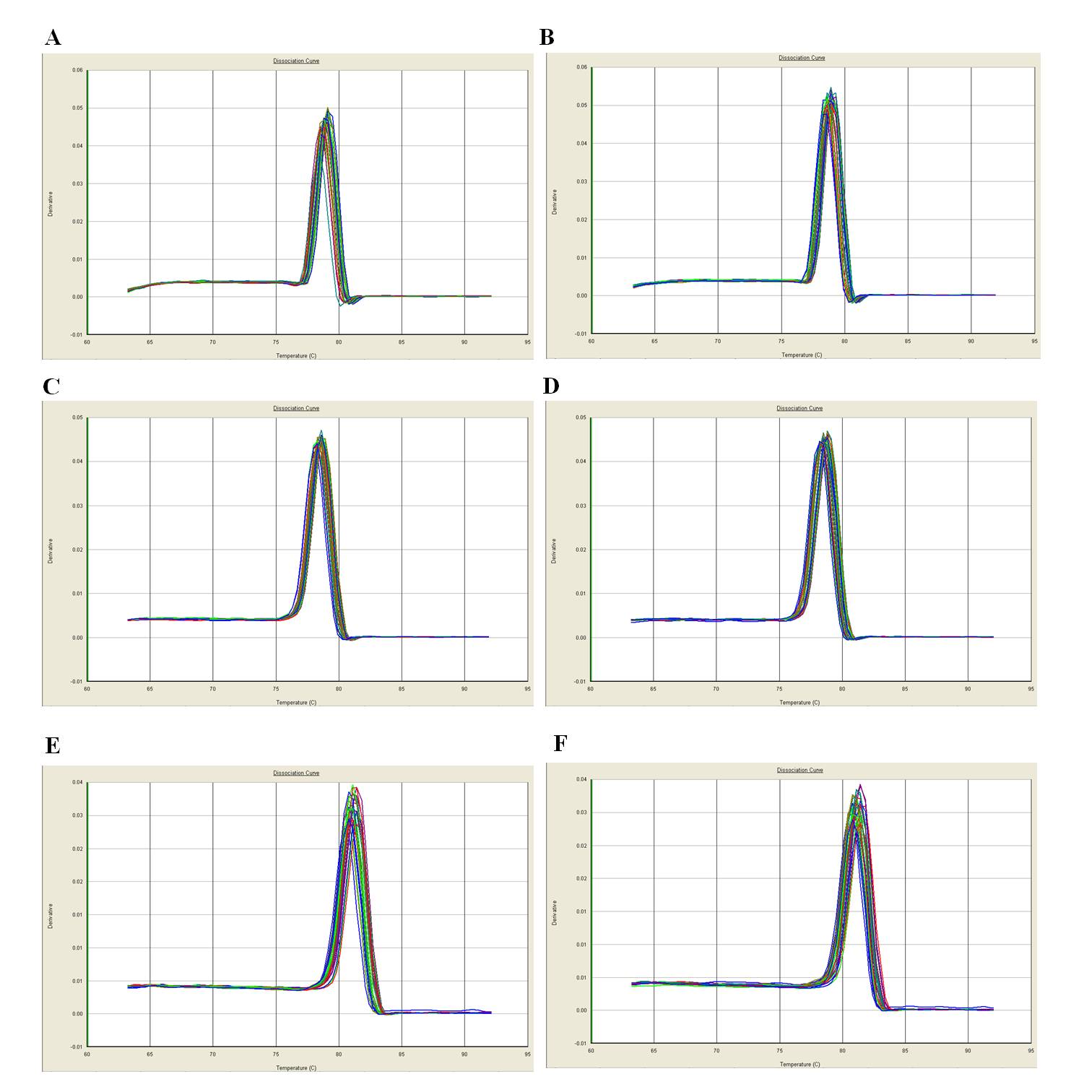

Supplement: Additional file 4 — Dissociation curves of ACT, MDH and TcPR-4b in Catongo (A, C, E) and TSH1188 (B, D, F), respectively. For all the harvesting point, the PCR amplification occurred at the same melting temperature showing that only the TcPR-4b gene was amplified. [file 1471-2229-14-161-S4.docx]
