# Supplementary material for: The pathogenesis-related protein PR-4b from Theobroma cacao presents RNase activity, Ca2+ and Mg2+ dependent-DNase activity and antifungal action on Moniliophthora perniciosa
Source: BMC Plant Biol. 2014 Jun 11;14:161. doi: 10.1186/1471-2229-14-161 (PMC4079191; doi:10.1186/1471-2229-14-161)
Supplement: Additional file 5 — RNase and DNase activity test using the extract of the bacteria containing the pET28 vector without insert (negative control), avoiding the possible action of some bacterial component on the obtained result. A. DNase activity. B. RNase activity. [file 1471-2229-14-161-S5.docx]

**Additional file 5.** RNase and DNase activity test using the extract of the bacteria containing the pET28 vector without insert (negative control), avoiding the possible action of some bacterial component on the obtained result. **A.** DNase activity. **B.** RNase activity.

**A**


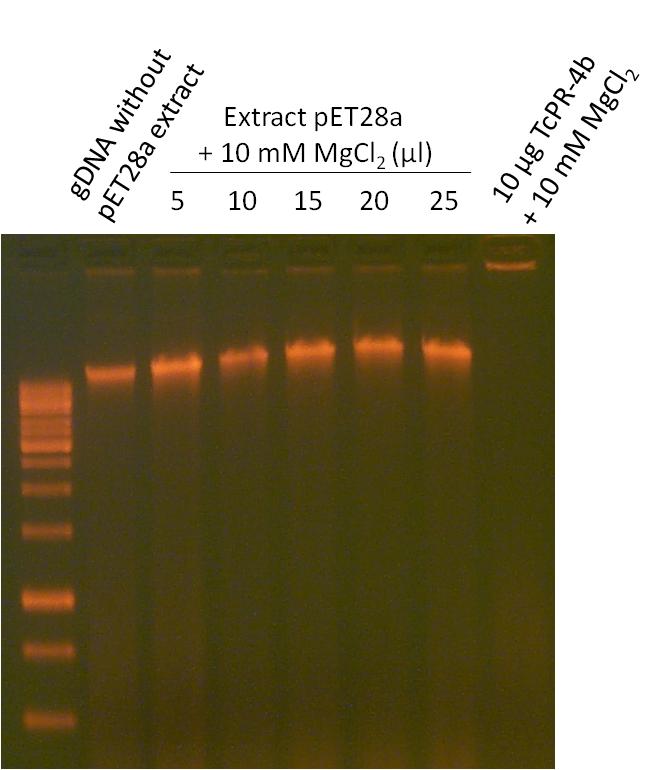


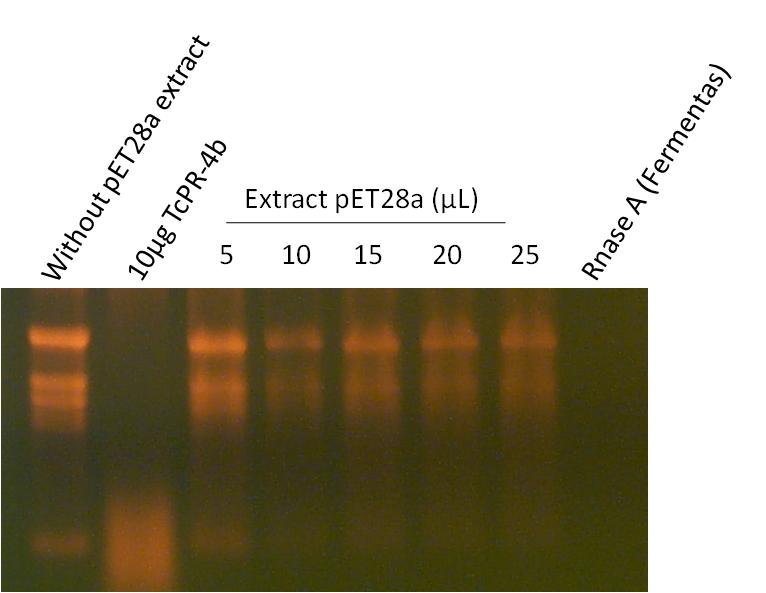


**B**
